# T136. DO VITAMIN D SUPPLEMENTATION DURING THE FIRST YEAR OF LIFE PREDICT COGNITION IN PSYCHOSES DURING MIDLIFE?

**DOI:** 10.1093/schbul/sby016.412

**Published:** 2018-04-01

**Authors:** Jussi Seppälä, Laura Tröger, Erika Jääskeläinen, Jouko Miettunen, Matti Isdohanni, Marianne Haapea

**Affiliations:** 1 Carea, Psychiatric Services; 2 University of Oulu

## Abstract

**Background:**

Schizophrenia (SCZ) has been associated with cognitive impairment.

The lack of vitamin D was associated with over 2-fold risk for mild cognitive impairment, and vitamin D could also associate with cognitive performance which may be explained by the role of vitamin D in the development of central nervous system or in neuroprotection. Vitamin D supplementation during the first year of life has been associated with a reduced risk of SCZ in males within the Northern Finland Birth Cohort 1966(NFBC 1966), but no studies have examined it`s possible association with cognition in SCZ during midlife.

The aim of this study was to examine the association of vitamin D supplementation during the first year of life with the cognition at the age of 43 years separately among those having psychosis and among non-psychotic controls in prospective NFBC 1966.

**Methods:**

The study is based on the NFBC 1966 concerning 12.058 live-born children in 1966 in Northern Finland. The final study population of this study (N= 257) consisted of 60 persons with schizophrenia spectrum disorder (SSD) and of 26 individuals with non-schizophrenic psychoses (NSSD) while 171 non-psychotic participants formed the reference group.

The daily dose of vitamin D was calculated based on the concentration of vitamin D in the product used and the reported amount of the product consumed. At the time when cohort was born, the recommended dose of vitamin D was 2000 IU/day. Based on maternal interviews in the year after birth, two measures of vitamin D supplementation were available: (a) frequency of intake (coded as regularly or irregularly/none) and (b) dose of vitamin D (<1600 IU/day, 1601–2000 IU/day, or >2000 IU/day.

The following tests were performed at the age of 43 years: California Verbal Learning Test (CVLT), Abstraction Inhibition and Working Memory Task (AIM), Visual Object Learning Test (VOLT), Vocabulary (WAIS-III), Visual Series (WMS-III), Digit Span (WAIS-III), Grooved Pegboard, Matrix Reasoning and Verbal Fluency.

**Results:**

The study population (N= 2579 included 60 subjects with SSD, 26 persons had NSSD, and 171 non-psychotic controls formed the reference group.

There were more men among those having psychosis (52.3% vs. 47.7%, respectively while the control group had more women (49.7 vs. 50.3, respectively).

Only 13.2% of participants in the entire study population had received vitamin D supplementation irregularly or not at all. On the other hand, 5.1% had taken vitamin D supplementation more than the recommended dose. Because the number of those who got vitamin supplementation under recommended dose (<2000IU/day) was not more than 3 persons (1.2% of the whole study population), the association of the dose vitamin D supplements with later cognition was not analyzed. Therefore, the frequency of vitamin D supplementation (coded as regular or irregular/none) was utilized in final analyses.

The frequency of vitamin D supplementation was not associated with cognition in midlife either among those having psychosis or in the control group (p-values for global cognitive performance in psychoses and controls were 0.89 and 0.61, respectively).

**Discussion:**

The main finding of this study was that no association was found between the frequency of vitamin D supplementation during first year of life and cognition in midlife either among those having psychosis or in the control group.

The only earlier study (N=9.114) evaluating a link between the use of nutritional supplements during early life and risk of SCZ was carried out in NFBC 1966. In males, the use of either irregular or regular vitamin D supplements was associated with a reduced risk of SCZ compared with no supplementation.

